# Higher fibroblast growth factor-23 concentrations associate with left ventricular systolic dysfunction in dialysis patients 

**DOI:** 10.5414/CN107991

**Published:** 2013-07-15

**Authors:** Shailendra Sharma, Jacob Joseph, Michel Chonchol, James S. Kaufman, Alfred K. Cheung, Zahi Rafeq, Gerard Smits, Jessica Kendrick, the HOST Investigators

**Affiliations:** 1 Division of Renal Diseases and Hypertension, University of Colorado Denver, Aurora, CO,; 2 Department of Medicine, Veterans Affairs Boston Healthcare System and Boston University School of Medicine,; 3 Harvard Medical School, Boston, MA,; 4 Medical Service, Veterans Affairs Salt Lake City Healthcare System,; 5 Division of Nephrology & Hypertension, University of Utah, Salt Lake City, UT,; 6 Department of Medicine, University of Massachusetts Medical School, Worcester, MA, and; 7 Denver Health Medical Center, Denver, CO, USA

**Keywords:** FGF-23, dialysis patients, LV dysfunction

## Abstract

Aims: The concentration of fibroblast growth factor 23 (FGF-23) is elevated in patients on dialysis. FGF receptors have been implicated in the pathogenesis of left ventricular (LV) hypertrophy. The objective of this study was to examine the associations between high plasma FGF-23 concentration and LV systolic dysfunction. Methods: We tested the hypothesis that high plasma FGF-23 concentration is associated with LV dysfunction in 110 chronic dialysis patients from the Homocysteine study who had paired echocardiograms performed for clinical indications. C-terminal FGF-23 concentrations were measured in stored plasma samples. Multivariate regression analyses were performed to evaluate the association of FGF-23 concentration with LV dysfunction. Results: Participants had a mean age of 60 ± 11 years. Median FGF-23 level and mean ejection fraction (EF) at baseline were 4,632 (1,384 – 14,997) RU/ml and 50 ± 13%, respectively. Median follow-up time was 1.9 years. Higher FGF-23 concentration was directly associated with decreases in EF during follow-up. After adjustment for demographics, baseline EF, hypertension, diabetes, cardiovascular disease, body mass index, systolic blood pressure, hemoglobin and markers of mineral metabolism, participants with FGF-23 in the highest tertile had an 8% decrease in EF compared to participants in the lowest tertile (β –8.0, 95% CI –15.5 to –0.53; p = 0.04). When FGF-23 was evaluated as a continuous variable, for every log10 increase in FGF-23, EF decreased during follow-up by 6.5% (β –6.5, 95% CI –11.3 to –1.73; p = 0.01). Conclusion: In conclusion, higher FGF-23 concentration is independently associated with LV systolic dysfunction in chronic dialysis patients.

## Introduction 

Estimates indicate that more than 20 million people have chronic kidney disease (CKD) in the United State [1]. CKD is associated with extraordinarily high rates of cardiovascular disease (CVD) and mortality [2, 3]. Although populations with advanced CKD have a high prevalence of traditional cardiovascular risk factors, the severity and extent of their CVD is disproportionate to these risk factor profiles [4]. Hence, there has been interest in identifying other cardiovascular risk factors in this patient population including regulators of phosphorus metabolism like fibroblast growth factor 23 (FGF-23). 

FGF-23 is a hormone that is secreted by osteocytes and osteoblasts. FGF-23 was initially regarded as a pathogenic factor primarily involved in hereditary hypophosphatemic rickets. Over the years, FGF-23 has been established as a regulator of circulating phosphate and 1,25-dihydroxyvitamin D (1,25(OH)_2_D) concentrations. FGF-23 promotes the urinary excretion of phosphorus and decreased intestinal absorption of phosphorus through inhibition of 1,25(OH)_2_D synthesis [5]. Thus, FGF-23 is a key regulator of phosphorus that maintains serum phosphorus within the normal range in patients with CKD. Serum FGF-23 concentrations increase early in the course of CKD, whereas serum phosphorus concentrations are usually only mildly increased or normal [6, 7, 8, 9]. By the time patients reach end stage renal disease, FGF-23 concentrations are often 100 times above the normal range [6]. 

Recently, elevated FGF-23 concentrations were found to be associated with death, cardiovascular events and kidney disease progression in patients with CKD [6, 7, 8, 9]. Left ventricular hypertrophy (LVH) is common [10], predisposes to left ventricular (LV) dysfunction, and is an important predictor of mortality in patients with CKD [11]. Elevated FGF-23 concentrations are independently associated with LVH in both the CKD and general population [12, 13, 14]. This suggests that FGF-23 is not only a novel risk factor for adverse outcomes in CKD but may also be a causal mechanism of LVH. While higher FGF-23 concentrations were strongly associated with an increased risk of mortality in a study of chronic dialysis patients [6], no studies to date have examined the relationship between plasma FGF-23 concentrations and LV function in chronic dialysis patients. We therefore conducted a longitudinal study to test the hypothesis that high plasma FGF-23 concentrations are associated with LV dysfunction independent of other factors that might influence systolic function in chronic dialysis patients utilizing the Homocysteine in Kidney and End Stage Renal Disease (HOST) Study [15] infrastructure. 

## Subjects and methods 

### Study cohort 

The details of the HOST study have been described previously [15]. Briefly, the HOST study was a multicenter, prospective, randomized, double-blind, placebo-controlled trial examining the effects of high doses of folic acid, pyridoxine hydrochloride (vitamin B_6_), and cyanocobalamin (vitamin B_12_) on death and cardiovascular events in patients with advanced kidney disease and elevated homocysteine concentrations. The trial enrolled 2,056 participants from 36 Veterans Affairs medical centers between September 2001 and October 2003. Patients were included in the study if they were 21 years of age or older with ESRD receiving either hemodialysis or peritoneal dialysis (n = 751), or with an estimated creatinine clearance (calculated by the Cockroft-Gault formula) of less than 30 ml/min but not yet on chronic dialysis (n = 1,305) and an elevated plasma homocysteine concentration of 15 µmol/l or higher. Participants were randomly assigned to receive a once-daily capsule containing 40 mg of folic acid, 100 mg of vitamin B_6_, and 2 mg of vitamin B_12_ or a daily placebo capsule. Each center’s institutional review board approved the study and all participants provided informed consent. 

### Echocardiograms 

For the current analysis, using the VA computerized patient record system, we identified a sub-group of 110 chronic dialysis patients who (i) were participants of the HOST study; (ii) had 2-dimensional transthoracic echocardiograms performed for clinical indications within 6 months before randomization in the HOST study; and (iii) had follow-up echocardiograms performed after randomization and during the course of the HOST study. All echocardiograms were performed on non-dialysis days. For each participant, we used paired echocardiograms to examine if baseline FGF-23 level was associated with change in LV EF over time. The echocardiogram performed before randomization was considered to be the baseline examination, while the echocardiogram performed during the course of the HOST study was to be the follow-up examination for the purpose of this analysis. Left ventricular mass indexed to body surface area was estimated by LV cavity dimension and wall thickness at end-diastole [16, 17]. Concentric and eccentric LVH was determined by calculation of the relative wall thickness (RWT). LVH was classified as concentric if RWT was > 0.42 and was classified as eccentric if RWT was ≤ 0.42 [16, 17]. 

### Laboratory measurements 

We used stored ethylene-diamine-tetra-acetate (EDTA) blood samples collected from the chronic dialysis participants 3 months after randomization for the measurement of FGF-23 concentrations. The HOST Executive Committee and the Cooperative Studies Program (CSP) of the Department of Veterans Affairs authorized the use of these plasma samples and these measurements. All laboratory measurements were performed at the Associated Regional University Pathologists (ARUP) Laboratories at the University of Utah, USA. 

C-terminal FGF-23 concentrations were measured in the plasma samples using a two-site second generation ELISA kit (Immutopics, San Clemente, CA, USA) with antibodies directed against two epitopes within the C-terminal region of the FGF-23 molecule. In addition to detecting intact FGF-23, this assay also detects its catabolic C-terminal fragments [18]. The analytical measurement range for the FGF-23 assay was 3.0 – 2,300 RU/ml. The coefficients of variation (CVs) were 2.6% and 1.4% at 32.1 and 299.2 RU/ml, respectively. The inter-assay CVs were 3.4% and 4.4% at 32.1 and 299.2 RU/ml, respectively. 

25-hydroxyvitamin D (25[OH]D) concentrations in the same plasma samples were measured by a commercial competitive chemiluminescent immunoassay on a Liaison analyzer, and 1,25-dihydroxyvitamin D (1,25(OH)_2_D) was measured by a commercial competitive radioimmunoassay (both from DiaSorin, Stillwater, MN, USA). The analytical measurement range for the 25(OH)D assay was 7 – 150 ng/ml. The intra-assay coefficients of variations (CVs) were 5.6% and 4.5% at 11 and 28 ng/ml, respectively. The inter-assay CVs were 9.1% and 5.6% at 16 and 51 ng/ml, respectively. For 1,25(OH)_2_D, the range of the assay was 5 – 200 pg/ml. The intra-assay CVs were 12.6% and 9.7% at 13 and 45 pg/ml, respectively. The inter-assay CVs were 21.4% and 14.7% at 25 pg/ml and 56 pg/ml, respectively. Intact parathyroid hormone (iPTH) was measured in the same plasma samples using a ROCHE E170 electrochemiluminescent immunoassay with a reference interval of 15 – 65 pg/ml. The intra-assay and inter-assay CVs were both less than 5%. 

### Other measurements 

Information collected at the time of randomization included a complete history and physical examination, demographics, health status, smoking status, etiology of kidney disease, history of hypertension, diabetes and CVD identified by self-report and chart review, and use of medications including angiotensin converting enzyme inhibitors (ACEI), angiotensin II receptor blockers (ARB), β-blockers and lipid lowering drugs. Systolic and diastolic blood pressures were measured in a standardized fashion [15]. Serum albumin, calcium and phosphorus were also measured at local sites using standard techniques. 

### Statistical analysis 

Only HOST study participants on chronic dialysis who underwent 2-dimensional echocardiograms at the specified time with stored EDTA-plasma for measurement of FGF-23 were included in the analysis. There were 110 patients who fulfilled these criteria. Wilcoxon rank-sum tests for continuous variables and Pearson χ^2^-test for categorical variables were used to compare demographic, cardiovascular disease risk factors, baseline LV EF and laboratory values including 25(OH)D, 1,25(OH)_2_D, and iPTH across tertiles of plasma FGF-23 concentrations. 

Tertiles of plasma FGF-23 concentrations were chosen as the primary predictor variable, with the lowest tertile serving as the reference group. In a separate analysis plasma FGF-23 concentrations were also evaluated as a continuous variable. Given the positively skewed distribution of FGF-23 concentrations, the values were transformed to the log base of 10. The association between plasma FGF-23 concentration and change in LV EF was analyzed using multivariable linear regression models. Covariates were included in the multivariable model if they were biologically plausible and were significantly correlated with abnormalities of mineral metabolism. Two sequential models were used. In Model 1, the variables included were age, gender, race, history of hypertension, diabetes and CVD, baseline LV EF, systolic blood pressure, body mass index (BMI), hemoglobin, phosphorus, plasma 25(OH)D, and 1,25(OH)_2_D. Model 2 contained all of the covariates in Model 1 plus the use of putative cardioprotective medications including angiotensin converting enzyme inhibitors (ACE inhibitors), angiotensin receptor blockers and β-blockers. The β-estimates and 95% confidence intervals were reported. Two-tailed values of p < 0.05 were considered statistically significant. All statistical analyses were performed with SAS software, version 9.13 (SAS Institute, Cary, NC, USA). 

## Results 

Baseline characteristics of the study population across FGF-23 tertiles are shown in Table 1. Of the 110 participants included in this analysis, the mean ± SD age of the participants was 60 ± 11 years, and 98% were male. The median (IQR) plasma FGF-23 level and mean LV EF at baseline were 4,632 (1,384 – 14,997) RU/ml and 50 ± 13%, respectively. There was no difference in time from randomization to measurement of FGF-23 concentration (Table 1), and the follow-up 2D-echocardiograms were performed after a median of 1.9 years from the plasma FGF-23 measurement. 

**Figure 1.**
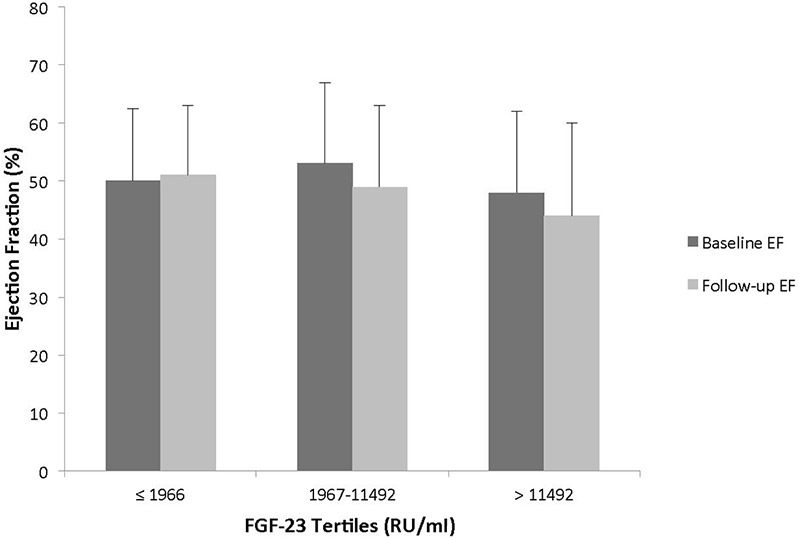
Ejection fraction at baseline and follow-up across tertiles of plasma FGF-23 concentrations. Ejection fraction measured at baseline and study follow-up according to tertiles of plasma FGF-23 concentrations in the 110 chronic dialysis patients.

Participants with FGF-23 concentrations in the highest tertile were younger and had higher iPTH and phosphate concentrations than subjects in the lowest tertile. There was no statistical difference in baseline LV EF, 1,25(OH)_2_D concentrations, or prevalent CVD across plasma FGF-23 tertiles. LV EF decreased in subjects with FGF-23 in the two highest tertiles but this did not reach statistical significance (p = 0.32) (Figure 1). Echocardiographic parameters at baseline and follow-up are shown in Table 2. Of note, 74.3% of subjects had LVH at baseline, of which the majority had concentric hypertrophy (Table 2). The mean LV EF of the entire cohort at the end of the follow-up period was 47 ± 14%. Importantly, no difference in the concentration of plasma FGF-23 was observed based on randomization to either the intervention group or the placebo group in the original HOST study. 

In linear multivariate regression models, higher FGF-23 concentrations were directly associated with a decrease in LV EF from the baseline to follow-up echocardiogram (Table 3). After adjustment for age, sex, race, history of hypertension, diabetes and CVD, baseline LV EF, BMI, systolic blood pressure, serum albumin, hemoglobin, phosphorus, plasma 25(OH) D and 1,25(OH)_2_D, participants with FGF-23 concentrations in the highest tertile had an 8% decrease in LV EF during follow-up, compared to participants in the lowest tertile (β –8.0, 95% CI –15.5 to –0.53; p = 0.04). Furthermore, when FGF-23 was evaluated as a continuous variable, for every log10 increase in FGF-23, LV EF during follow-up decreased by 6.5% (β –6.5, 95% CI –11.3 to –1.73; p = 0.01) after multivariate adjustment. Results were unchanged after adjustment for putative cardioprotective medications including β-blockers, ACE inhibitors and angiotensin receptor blockers. In addition, we tested for an interaction between logFGF-23 and baseline LVMI on LV EF and also tested for an interaction between log FGF-23 and baseline LVH on LV EF. We found that the association of FGF-23 with LV EF is the same independent of baseline LVMI and LVH (p for interaction > 0.20 for all). 

We also examined the association between log FGF-23 concentrations and follow-up LVMI. Although this relationship did not reach statistical significance, there was a trend towards an association between higher FGF-23 concentrations and higher LVMI (β 12.06, 95% CI 0.14 to 24.1; p = 0.05 per log_10_FGF-23 after adjustment for age, sex, race, diabetes, hypertension, CVD, systolic blood pressure, serum albumin, hemoglobin, phosphorus, plasma 25(OH)D and 1,25(OH)_2_D). We did not find any association between FGF-23 concentrations and LVH in this cohort (results not shown). 

In contrast to FGF-23, serum phosphate concentrations were not associated with LV EF in univariate or multivariate analyses. There was also no association between plasma 1,25(OH)_2_D concentrations with LV EF (for every log10 increase in 1,25(OH)_2_D: fully adjusted β –1.4, 95% CI –14.4 to 11.6, p = 0.84). Plasma 25(OH)D and iPTH concentrations were not associated with LV EF (results not shown). 

## Discussion 

In our current prospective cohort of chronic dialysis patients, we found higher plasma FGF-23 concentrations to be directly associated with a decrease in LV EF during the study follow-up. This association was not confounded by established risk factors known to cause lower LV EF. After adjustment for variables that can independently affect LV EF, high FGF-23 concentrations were negatively correlated with EF during follow-up. To our knowledge this is the first study examining the association between FGF-23 and left ventricular dysfunction in hemodialysis patients. 

Abnormal LV geometry is the leading cardiovascular condition in patients with CKD. The prevalence of LV dysfunction increases as kidney function declines. At the time of initiation of dialysis, the prevalence of LV dysfunction or overt LVH is estimated to be at least 75% [19]. In the non-CKD population with congestive heart failure (CHF), evidence from randomized trials suggests that treatment with certain medications including ACE inhibitors, angiotensin receptor blockers and β-blockers reduces morbidity and mortality in congestive heart failure (CHF). However, in the dialysis population, data regarding treatment of CHF is scarce and only carvedilol has been shown to improve LV function and decrease morbidity and mortality in a small cohort [20]. Hence, other treatments for CHF are needed in this high-risk population. Our findings of an independent association between high plasma FGF-23 concentrations and systolic dysfunction suggest that targeting disordered phosphorus metabolism may be a treatment strategy for CHF in dialysis patients. 

Previous studies have found an association between FGF-23 concentrations and LVH in patients with and without CKD [12, 13, 14]. In a study of 162 patients with CKD, FGF-23 concentrations were independently associated with LV hypertrophy and increased LV mass index (LVMI) [12]. Similar results were reported in a cohort of elderly patients with normal kidney function [14]. However, both of these cohorts purposely excluded subjects with LV dysfunction. Seiler et al. [18] recently evaluated the relationship between FGF-23 and LV dysfunction in 885 patients with relatively normal kidney function (mean eGFR 76.3 ± 19.4 ml/min/1.73 m^2^) undergoing elective coronary angiography in the HOM SWEET HOMe study. FGF-23 was inversely associated with EF after multivariate adjustment for age, race, gender, diabetes, eGFR, serum C-reactive protein concentrations, LVH, and the use of β-blockers and ACE inhibitors (β –2.03; p = 0.004) [21]. However, in contrast to our study, Seiler et al. did not adjust for other variables of mineral metabolism including 1,25(OH)_2_D and iPTH, which may be important confounders in the relationship between FGF-23 and adverse clinical outcomes. In our study, FGF-23 was associated with LV dysfunction even after adjustment for 1,25(OH)_2_D and iPTH, suggesting that the relationship between FGF-23 and LV dysfunction is independent of the vitamin D axis. 

The mechanism by which FGF-23 results in LV dysfunction is unknown. Elevated levels of FGF-23 have been linked to greater risks of LVH and mortality in patients with CKD, but whether these risks represent causal effects of FGF-23 is unknown. FGF-23 may simply be a biomarker of high phosphorus burden or it may have direct toxic effects on the myocardium. Elevated serum phosphate levels are associated with LVH in patients with and without CKD and in patients on dialysis, correction of hyperphosphatemia results in improvements in LVH [22, 23, 24]. One of the limitations of these studies is that FGF-23 was not measured, so it is unclear whether LVH would have been more strongly associated with FGF-23 than phosphate. Low 1,25(OH)_2_D concentrations have also been associated with LVH, and animal models have shown that administration of 1,25(OH)_2_D results in reductions in LVH [25, 26]. However, a recent study in CKD patients did not find any improvement in LVMI after administration of the active vitamin D analogue, paricalcitol [27]. In our study, only FGF-23 and neither plasma phosphate nor 1,25(OH)_2_D were associated with LV dysfunction, suggesting that FGF-23 may be acting directly on the myocardium. Experimental studies suggest that FGF-23 may have direct cardiac toxicity. A recent study found that FGF-23 directly induced LVH in mice [28]. Additionally, these investigators found that in an established animal model of CKD, treatment with an FGF-receptor blocker decreased LVH independently of blood pressure [28]. Thus, FGF-23 may play a direct role in the pathogenesis of LVH, which might progress to LV dysfunction and therefore may be a potential target for treatment. 

Our study has several limitations. First, given that this is an observational study, a causal relationship between higher FGF-23 concentrations and LV dysfunction cannot be established. Second, echocardiograms were performed for clinical indications. Thus, the dialysis patients in whom echocardiograms were performed might have had a larger cardiovascular disease burden than those not undergoing echocardiograms; however this potential confounding did not explain the relationship between FGF-23 concentrations and LV dysfunction. Third, we do not have any data on intercurrent clinical events such as myocardial infarctions between echocardiograms. Hence, it is possible that such an event occurred that changed LV EF through a mechanism independent of FGF-23 concentrations. Fourth, information on intradialytic weight gain, physical activity level and use of antioxidants is lacking among participants. Fifth, we did not have information on the use of nutritional vitamin D supplements or active vitamin D analogues by the patients. However, given the era in which the HOST study was performed, nutritional vitamin D supplementation was not prevalent in the chronic dialysis population. Finally, this study included mainly male dialysis patients, and caution should be used when extrapolating these results to other patient populations. 

The present study also has several strengths. First, to our knowledge this is the first study reporting a link between plasma FGF-23 concentrations and LV dysfunction in patients undergoing maintenance dialysis. Second, we were able to adjust for established risk factors for LV dysfunction and variables of mineral metabolism including 25(OH)D and 1,25(OH)_2_D. Finally, all FGF-23 measurements were performed in a standardized fashion. 

In conclusion, we found that high plasma FGF-23 concentrations were a strong and independent predictor of LV systolic dysfunction in chronic dialysis patients. This finding should be corroborated by studies where echocardiograms are protocol-based rather than being performed for clinical indication. Further studies are needed to determine the mechanisms by which higher FGF-23 concentrations are associated with LV dysfunction and whether therapeutic interventions to lower FGF-23 concentrations improve LV systolic function in dialysis patients. 

## Acknowledgments 

The research reported in this study was supported by the Department of Veterans Affairs Cooperative Studies Program and the HOST Executive Committee (Drs. Rex L. Jamison, Pamela Hartigan, James Kaufman, David S. Goldfarb, Stuart R. Warren, Peter D. Guarino, and J. Michael Gaziano). Additional support was provided by the National Institute of Diabetes and Digestive and Kidney Diseases K23 DK087859-01A1, RO1 DK 081473, and RO1 DK 078112, and an AMGEN fellowship grant. 

## Financial support 

National Institute of Diabetes and Digestive and Kidney Disease grants K23 DK087859-01A1, RO1 DK 081473, and RO1 DK 078112 as well as an Amgen fellowship grant. 

## Disclosure 

Dr. Cheung is a consultant for Amgen and Baxter. The other authors do not have any interests to disclose. 

**Table 1.** Baseline characteristics of study participants by tertiles of baseline plasma FGF-23 concentrations.

Characteristic	FGF-23 (RU/ml)			p-value
	≤ 1,966	1,967 – 11,492	> 11,492	
Age (years)	61 ± 11	63 ± 12	57 ± 11	0.08
BMI (kg/m^2^)	26.3 ± 4.1	27.2 ± 4.9	25.8 ± 3.8	0.51
Systolic blood pressure (mmHg)	142.4 ± 21.5	143.0 ± 28.7	135.0 ± 24.8	0.47
Serum hemoglobin (g/dl)	12.0 ± 1.6	11.7 ± 1.8	11.8 ± 1.5	0.67
Serum albumin (g/dl)	3.8 ± 0.4	3.8 ± 0.4	3.8 ± 0.5	0.86
Plasma iPTH (pg/ml)	184 ± 153	257 ± 216	466 ± 456	0.0003
Serum calcium (mg/dl)	9.0 ± 0.7	9.1 ± 0.8	9.4 ± 0.7	0.09
Serum phosphorus (mg/dl)	5.0 ± 1.6	5.5 ± 1.7	6.4 ± 1.4	0.0008
Plasma 25(OH)D (ng/ml)	16.9 ± 9.7	19.5 ± 12.7	16.9 ± 9.6	0.84
Plasma 1,25(OH)_2_D (pg/ml)	13.2 ± 8.2	14.7 ± 8.5	16.4 ± 17.7	0.43
Serum total cholesterol (mg/dl)	157.8 ± 37.4	151.8 ± 38.7	143.9 ± 35.8	0.19
Serum HDL-C (mg/dl)	43.4 ± 12.6	40.7 ± 13.4	37.6 ± 10.1	0.15
Serum LDL-C (mg/dl)	85.0 ± 29.1	85.9 ± 35.6	78.8 ± 29.4	0.51
Serum triglycerides (mg/dl)	163.2 ± 174	149.2 ± 106	154.1 ± 117	0.90
Serum baseline LV EF (%)	50.3 ± 12.5	52.5 ± 13.6	47.5 ± 13.9	0.32
Time on dialysis (months)	21.9 ± 34.5	16.1 ± 19.5	56.4 ± 62.2	< 0.0001
Time from randomization to measurement of FGF-23 (days)	106.3 ± 54.4	94.4 ± 8.5	92.9 ± 11.3	0.45

All values are reported as mean ± SD unless otherwise stated. FGF-23 = fibroblast growth factor 23; BMI= body mass index; iPTH = intact parathyroid hormone; 25(OH)D = 25-hhydroxyvitamin D; 1,25(OH)_2_D = 1,25-dihydroxyvitamin D; HDL-C = high density lipoprotein cholesterol; LDL-C = low density lipoprotein cholesterol; LV = left ventricular; EF = ejection fraction.

**Table 2.** Echocardiographic parameters at baseline and follow-up.

Parameters	Baseline	Follow-up
Left ventricular ejection fraction (%)	48.6 ± 12.4	46.5 ± 14.1
Left ventricular mass index (g/m^2^)	128.8 ± 42.2	129.4 ± 36.9
Left ventricular end diastolic diameter (mm)	5.22 ± 0.73	5.3 ± 0.81
Intraventricular septal thickness at end-diastole (mm)	1.22 ± 0.34	1.21 ± 0.26
Posterior wall thickness at end-diastole (mm)	1.21 ± 0.26	1.19 ± 0.24
Left ventricular hypertrophy (%)		
Yes	74.3	77.7
No	25.7	22.3
Concentric LVH (%)	61.5	65
Eccentric LVH (%)	38.5	35

All values are expressed as mean ± standard deviation unless otherwise specified. LVH = left ventricular hypertrophy.

**Table 3.** Relationship between plasma FGF-23 concentrations and change in left ventricular ejection fraction during follow-up in chronic dialysis patients.

Model	FGF-23 tertiles (RU/ml) difference (95% CI)			Log_10_ FGF-23 difference (95% CI)
	≤ 1,966	1,967 – 11,492	> 11,492	
Unadjusted	0.0 (REF)	–1.0 (–7.2 to 5.2)	–6.7 (–13.1 to –0.33)	–4.3 (–9.3 to 0.66)
Model 1	0.0 (REF)	0.13 (–6.6 to 6.9)	–8.0 (–15.5 to –0.53)	–6.5 (–11.3 to –1.73)
Model 2	0.0 (REF)	0.12 (–6.8 to 7.0)	–8.4 (–16.2 to –0.62)	–7.2 (–12.3 to –2.1)

Model 1: adjusted for age, gender, race, diabetes, hypertension, history of cardiovascular disease, body mass index, systolic blood pressure, serum albumin, hemoglobin, phosphorus, plasma 25-hydroxyvitamin D and 1,25-dihydroxyvitamin D. Model 2: adjusted for covariates in Model 1 plus use of angiotensin converting enzyme inhibitors, angiotensin receptor blockers and β-blockers.
